# The specific core fucose-binding lectin *Pholiota squarrosa* lectin (PhoSL) inhibits hepatitis B virus infection in vitro

**DOI:** 10.1038/s41598-023-28572-6

**Published:** 2023-04-15

**Authors:** Tsunenori Ouchida, Haruka Maeda, Yuka Akamatsu, Megumi Maeda, Shinji Takamatsu, Jumpei Kondo, Ryo Misaki, Yoshihiro Kamada, Masahiro Ueda, Keiji Ueda, Eiji Miyoshi

**Affiliations:** 1 Department of Molecular Biochemistry and Clinical Investigation, Osaka University Graduate School of Medicine, 1-7 Yamada-Oka, Suita, Osaka 565-0871 Japan; 2 Laboratory of Single Molecule Biology, Graduate School of Frontier Biosciences, Osaka University, Suita, Osaka 565-0871 Japan; 3 Division of Virology, Department of Microbiology and Immunology, Osaka University Graduate School of Medicine, Suita, Osaka 565-0871 Japan; 4 Applied Microbiology Laboratory, International Center for Biotechnology, Osaka University, Suita, Osaka 565-0871 Japan; 5 Department of Advanced Metabolic Hepatology, Osaka University Graduate School of Medicine, Suita, Osaka 565-0871 Japan

**Keywords:** Infection, Hepatology, Hepatitis B virus, Glycosylation

## Abstract

Glycosylation of proteins and lipids in viruses and their host cells is important for viral infection and is a target for antiviral therapy. Hepatitis B virus (HBV) is a major pathogen that causes acute and chronic hepatitis; it cannot be cured because of the persistence of its covalently closed circular DNA (cccDNA) in hepatocytes. Here we found that *Pholiota squarrosa* lectin (PhoSL), a lectin that specifically binds core fucose, bound to HBV particles and inhibited HBV infection of a modified human HepG2 cell line, HepG2-hNTCP-C4, that expresses an HBV receptor, sodium taurocholate cotransporting polypeptide. Knockout of fucosyltransferase 8, the enzyme responsible for core fucosylation and that aids receptor endocytosis, in HepG2-hNTCP-C4 cells reduced HBV infectivity, and PhoSL facilitated that reduction. PhoSL also blocked the activity of epidermal growth factor receptor, which usually enhances HBV infection. HBV particles bound to fluorescently labeled PhoSL internalized into HepG2-hNTCP-C4 cells, suggesting that PhoSL might inhibit HBV infection after internalization. As PhoSL reduced the formation of HBV cccDNA, a marker of chronic HBV infection, we suggest that PhoSL could impair processes from internalization to cccDNA formation. Our finding could lead to the development of new anti-HBV agents.

## Introduction

Hepatitis B virus (HBV) is a major pathogen that causes acute and chronic hepatitis. About 300 million individuals worldwide were chronically infected with HBV in 2019^1^. People infected with HBV can develop liver cirrhosis and hepatocellular carcinoma^2^. Current anti-HBV therapies include nucleoside and nucleotide analogs, which can competitively inhibit HBV replication^2,3^, and pegylated interferons, which can modulate the host immune response to HBV infection and induce the degradation of covalently closed circular DNA (cccDNA) in hepatocytes^4^. However, long-term treatment with nucleoside analogs can select drug-resistant viruses, and interferons can present severe adverse effects with low tolerability.

It is known that HBV binds to heparan sulfate on hepatocytes as the first step of infection. In 2012, sodium taurocholate cotransporting polypeptide (NTCP) was identified as an essential receptor for HBV and hepatitis delta virus^5^. NTCP was originally identified as an integral membrane glycoprotein that functions in the enterohepatic circulation of bile acids. Glycosylation of NTCP is involved in its biological function^6^. Glycosylation is also a target of antiviral therapy^7,8^.

Many cell-surface proteins, including virus receptors and viral-envelope proteins, are glycoproteins. Changes in the glycosylation status of proteins can affect virus particle endocytosis and infection^9^. For example, the cleavage of sialic acids to specific proteins by sialidase on host cells is required for influenza infection^10^; inhibition of sialidase is a target for anti-influenza therapy^7^. Sialylation of host cells is also required for infection by SARS-CoV-2^11^.

One of our previous studies used oligosaccharide-remodeling cells to demonstrate that core fucosylation is the key glycosylation step required for entry of pseudo-HBV particles (also known as bio-nano capsules) into hepatoma cells^12^. Alpha-(1,6)-fucosyltransferase (FUT8) is the unique fucosyltransferase responsible for core fucosylation of N-glycans^13^. As the core fucose is involved in receptor endocytosis^14–16^, knockdown of *FUT8* or upregulation of *FUT8* expression changed the level of bio-nano capsule incorporation into hepatoma cells. We also observed an association of NTCP with a scaffold protein, myosin-9, together with an increase in core fucose levels in hepatoma cells. However, the later study revealed differences in the infection process between HBV particles and bio-nano capsules. Whereas bio-nano capsules only bind to heparan sulfate on the cell surface^17^, HBV particles bind to both heparan sulfate and NTCP.

We also previously found that *Pholiota squarrosa* lectin (PhoSL) binds to core fucoses and is stable under high temperatures and acidic conditions^18^. Many lectins, proteins that bind specifically to glycans, are antiviral agents^19^. For example, the lectins griffithsin, cyanovirin-N, and scytovirin bind to mannose-rich oligosaccharides and can inhibit human immunodeficiency virus (HIV)-1 infection without cytotoxicity^20–22^. Cyanovirin-N has also been studied in the preclinical phase of drug development^8^.

In this study, we investigated the effects of PhoSL on HBV infection of the human cell line HepG2-hNTCP-C4. We also analyzed the molecular mechanisms underlying PhoSL-mediated inhibition of HBV infection. Core fucosylation of cellular proteins was important for HBV infection, and PhoSL inhibited HBV entry from internalization to cccDNA formation by binding to HBV itself and by blocking the activation of epidermal growth factor receptor (EGFR), which usually enhances HBV infection.

## Results

### PhoSL inhibits HBV infection of HepG2-hNTCP-C4 cells

**Figure 1:**
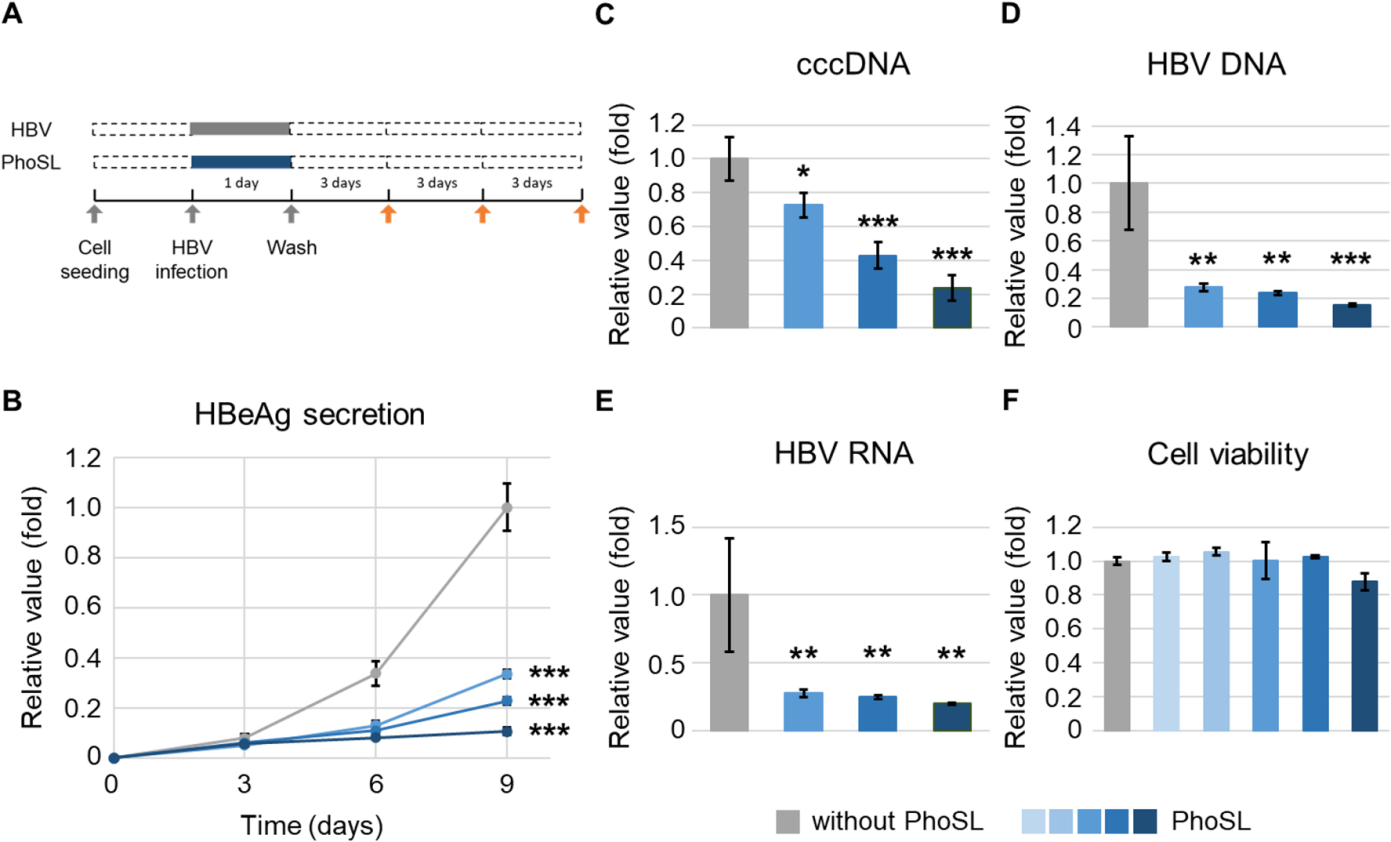
PhoSL inhibited HBV infection in a dose-dependent manner. (**A**) Time course of the HBV infection assay. Addition of PhoSL occurred at the same time as HBV inoculation. The orange arrows indicate the collection of conditioned medium. (**B**) HBeAg ,the antigen usually tested to monitor HBV infection, secretion from HepG2-hNTCP-C4 cells in the presence or absence of PhoSL was measured by ELISA. HepG2-NTCP-C4 cells were infected with HBV particles and treated with PhoSL (0, 2.5, 5, or 10 μg/mL). Some of the conditioned medium from each treatment was collected on day 3, day 6, and day 9 (day 0 was the day of washing after HBV inoculation). The increasing gradient of blue color represents PhoSL concentrations of 2.5, 5, and 10 μg/mL, respectively. Gray indicates 0 μg/mL PhoSL. Dunnett’s test, ***P < 0.001 vs. 0 μg/mL PhoSL at day 9. Error bars show SD; n = 3. (**C**, **D**, **E**) cccDNA, HBV DNA, and HBV RNA were extracted and purified from HBV-infected HepG2-NTCP-C4 cells at day 9 and were quantified by qPCR. (**C**) and (**D**) show the copy numbers of cccDNA and HBV DNA calculated by absolute quantification, respectively. (**E**) shows the values of HBV RNA normalized to *GAPDH* mRNA. The increasing gradient of blue color represents PhoSL concentrations of 2.5, 5, and 10 μg/mL, respectively. Gray indicates 0 μg/mL PhoSL. Dunnett’s test, *P < 0.05, **P < 0.01, ***P < 0.001 vs. 0 μg/mL PhoSL. Error bars show SD; n = 3. (**F**) Cell viability of HepG2-NTCP-C4 cells cultured for 24 h with PhoSL. The increasing gradient of blue color represents PhoSL concentrations of 0.5, 1, 2.5, 5, and 10 μg/mL, respectively. Gray indicates 0 μg/mL PhoSL. Live cells were measured with the CellTiter-Glo Luminescent Cell Viability Assay kit. Dunnett’s test was performed. There were no statistically significant differences between 0.5, 1, 2.5, 5, and 10 μg/mL PhoSL vs. 0 μg/mL PhoSL. Error bars show SD; n = 3.

First, we examined whether PhoSL inhibited HBV infection of HepG2-hNTCP-C4 cells. HepG2-hNTCP-C4 cells are a clonal cell line established from the human hepatoma cell line HepG2. HepG2-hNTCP-C4 cells overexpress hNTCP, a receptor of HBV, and are susceptible to HBV infection^23^. HepG2-hNTCP-C4 cells were inoculated with HBV in the presence or absence of PhoSL at several concentrations for 1 day. After removal of free HBV and PhoSL, the cells were cultured for 9 days (Fig. 1A). Treatment with PhoSL dramatically decreased the levels of human HBV e antigen (HBeAg) (Fig. 1B), cccDNA (Fig. 1C), HBV DNA (Fig. 1D), and HBV RNA (Fig. 1E), markers of HBV infection, in a dose-dependent manner. Then, we checked whether PhoSL was cytotoxic to HepG2-hNTCP-C4 cells using the CellTiter-Glo Luminescent Cell Viability Assay kit (Promega), which determines the number of viable cells in culture based on quantitation of adenosine 5′-triphosphate. PhoSL (0.5–10 μg/mL) did not affect cell viability (Fig. 1F). These data suggest that PhoSL can inhibit HBV infection without cytotoxicity.

### PhoSL blocks EGFR activation, which inhibits HBV infection

**Figure 2:**
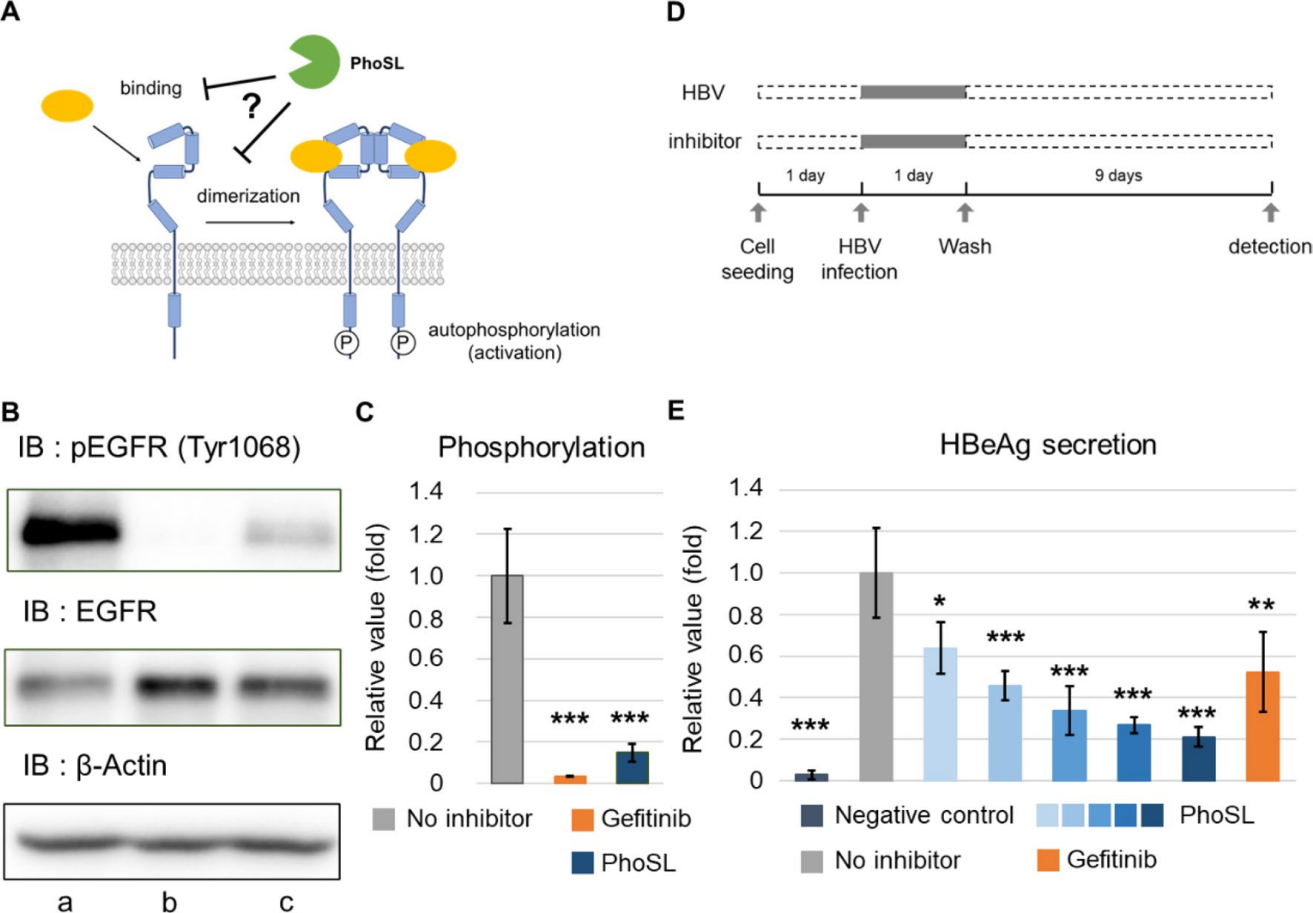
PhoSL blocked EGFR activation. (**A**) The hypothesis of PhoSL blockade of EGFR activation. EGFR is activated after EGF binding, dimerization, and oligomerization. (**B**) Phosphorylated EGFR in cell lysates was detected by immunoblot. After starvation in serum-free medium, HepG2-hNTCP-C4 cells were stimulated with EGF (100 ng/mL) with gefitinib (10 μM) or PhoSL (10 μg/mL) at 37 °C for 10 min. a: no inhibitor; b: gefitinib (10 μM); c: PhoSL (10 μg/mL). Representative blots are shown. The original blot images are available in Supplementary Fig. S3 online. (**C**) Quantification of immunoblot results from (**B**). The relative values of pEGFR (Tyr1068) to EGFR were calculated using Fiji software and were normalized to the mean of the “no inhibitor” samples. The quantified areas are shown in Supplementary Fig. S3 online. Dunnett’s test, ***P < 0.001 vs. no inhibitor. Error bars show SD; n = 3. (**D**) Time course of HBV infection with gefitinib or PhoSL. (**E**) HBeAg secretion from HepG2-hNTCP-C4 cells infected with HBV particles in the presence of gefitinib (10 μM) or PhoSL at various concentrations was measured by ELISA. The values were normalized by the mean of the “no inhibitor” samples. The increasing gradient of blue color represents PhoSL concentrations of 0.5, 1, 2.5, 5, and 10 μg/mL, respectively. Gray indicates 0 μg/mL PhoSL. Dunnett’s test, *P < 0.05, **P < 0.01, ***P < 0.001 vs. no inhibitor. Error bars show SD; n = 3.

To determine how PhoSL inhibits HBV infection, we considered two possibilities: (1) PhoSL affects the protein dynamics of the host cells, or (2) PhoSL binds to HBV. First, we tested whether PhoSL inhibits HBV infection by affecting protein activity in host cells. EGFR activation enhances HBV entry^24^. EGFR is a heavily N-glycosylated protein, and core fucosylation affects the high-affinity binding of epidermal growth factor (EGF) to EGFR^25^. EGFR is activated by EGF binding, receptor dimerization, autophosphorylation of its intracellular domain, and oligomerization. Thus, we hypothesized that PhoSL inhibits HBV infection by blocking EGFR activation (Fig. 2A). We measured EGFR phosphorylation as a measure of its activation in the presence or absence of PhoSL. After cell seeding and culture for 1 day, HepG2-hNTCP-C4 cells were starved in serum-free medium for 1 day. Then, the cells were pretreated with PhoSL or gefitinib (an inhibitor of EGFR activation) and stimulated with EGF (100 ng/mL). PhoSL blocked EGFR phosphorylation as measured by immunoblot (Fig. 2B,C). Next, we investigated whether PhoSL could block EGFR activation by inhibiting the binding of EGF to EGFR. We treated HepG2-hNTCP-C4 cells with EGF-tetramethylrhodamine conjugate (EGF-TAMRA). Unfortunately, despite EGF-TAMRA-mediated activation of EGFR detected by immunoblot, we did not observe EGF-TAMRA bound to the cells by confocal microscopy (Supplementary Fig. S1 online). Because HepG2 cells express low levels of EGFR^26^, we tested EGF-TAMRA binding to a human pancreatic cancer cell line, PANC-1, which expresses high levels of EGFR. PANC-1 cells are core fucosylated like HepG2-hNTCP-C4 cells are, and PANC-1 cells expressed higher levels of EGFR than HepG2-hNTCP-C4 cells (Supplementary Fig. S2 online). Using confocal microscopy, we observed EGF-TAMRA bound to PANC-1 cells, and PhoSL blocked EGF-TAMRA binding (Supplementary Fig. S2 online). These data indicate that PhoSL inhibits EGFR activation by blocking EGF binding to EGFR. Finally, to compare the effect of PhoSL with that of gefitinib on HBV infection, HepG2-hNTCP-C4 cells were inoculated with HBV particles in the presence of PhoSL or gefitinib (Fig. 2D). Interestingly, although the inhibition of EGFR phosphorylation by PhoSL was weaker than by gefitinib, the level of secreted HBeAg after treatment with PhoSL was lower than that of gefitinib (Fig. 2E). These results suggest that PhoSL can inhibit HBV infection by blocking EGFR activation.

### PhoSL inhibits HBV infection in HepG2-hNTCP-C4 WT and *FUT8* KO cells

**Figure 3:**
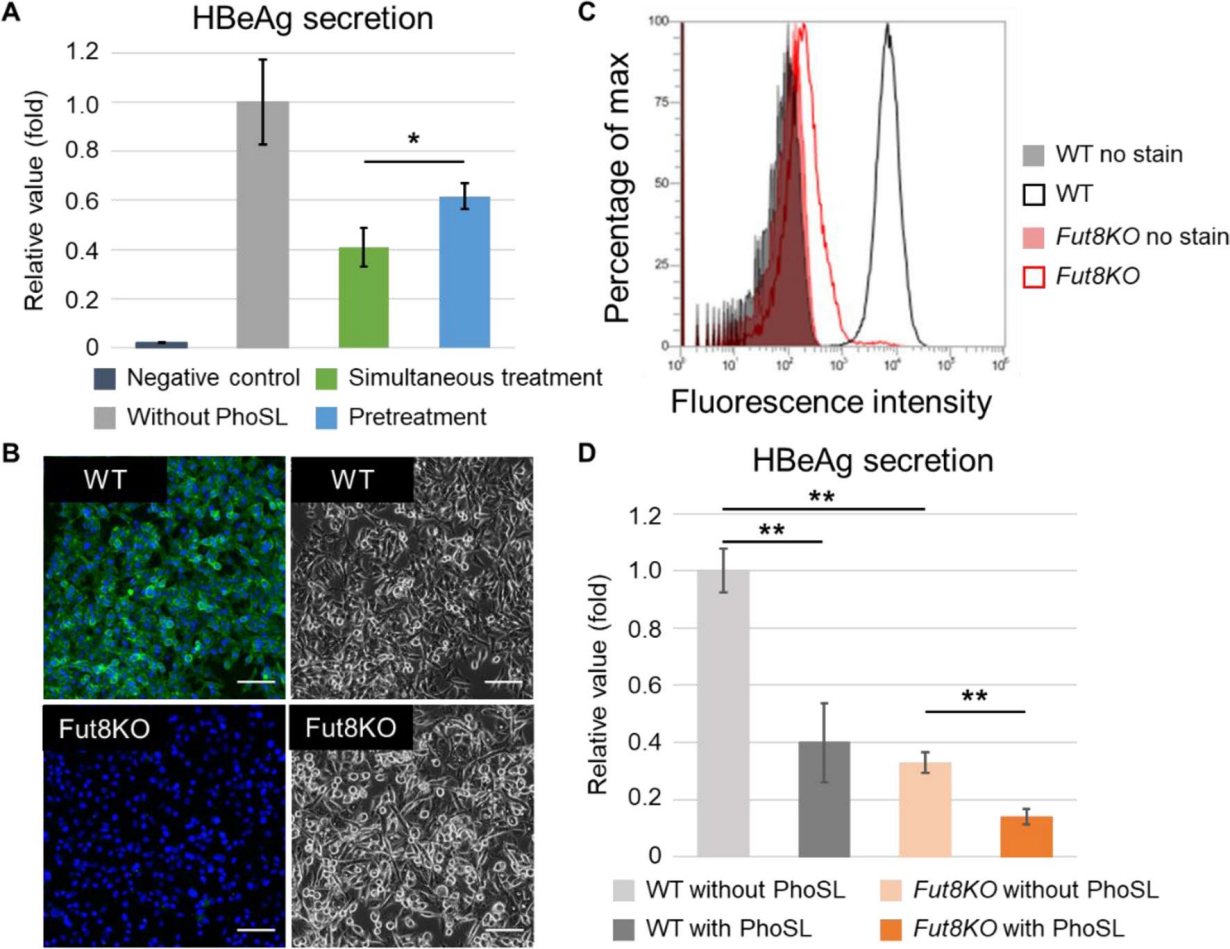
PhoSL inhibited HBV infection of HepG2-hNTCP-C4 WT and *FUT8* KO cells. (**A**) HBeAg secretion from HepG2-hNTCP-C4 cells treated with PhoSL and infected with HBV particles was measured by ELISA. The cells were pretreated with PhoSL at 4 °C for 1 h before inoculation with HBV particles, simultaneous treatment with PhoSL and HBV particles, or inoculation with HBV particles in the absence of PhoSL. The values were normalized to the mean of HBeAg secretion levels in the absence of PhoSL. Student’s t test, *P < 0.05. Error bars show SD, n = 3. (**B**) HepG2-hNTCP-C4 WT and *FUT8* KO cells were stained with PhoSL-FITC and observed using confocal microscopy (left). Green: PhoSL-FITC; blue: nucleus (Hoechst 33,342 stain). Bright-field images are shown on the right. Scale bars show 100 μm. (**C**) Flow cytometry of HepG2-hNTCP-C4 WT and *FUT8* KO cells with or without PhoSL-FITC. Black: WT; red: *FUT8* KO. Solid lines without fill show the cells stained with PhoSL-FITC. Filled areas show unstained cells. (**D**) HBeAg secretion from HepG2-hNTCP-C4 WT and *FUT8* KO cells infected with HBV particles in the absence or presence of PhoSL (10 μg/ml) was measured by ELISA. The values were normalized to the mean of WT cells in the absence of PhoSL treatment. Black: WT; orange: *FUT8* KO. The darker colors show the HBeAg secretion levels from cells treated with PhoSL. The lighter colors show the HBeAg secretion levels from cells not treated with PhoSL. Student’s t test with Bonferroni correction, **P < 0.01, vs. no PhoSL. Error bars show SD, n = 3.

Next, we examined whether PhoSL inhibits HBV infection by binding to HBV . We compared HBV infection of HepG2-hNTCP-C4 wild-type (WT) cells with PhoSL pretreatment (at 4 °C for 1 h) or by simultaneous treatment with PhoSL and HBV particles. After culturing the treated and inoculated cells for 9 days, we found that HBeAg secretion levels from cells simultaneously treated with HBV and PhoSL were lower than from cells pretreated with PhoSL before HBV inoculation (Fig. 3A). In addition, we investigated whether PhoSL inhibited HBV infection of core fucosylation-deficient HepG2-hNTCP-C4 cells. FUT8 is responsible for core fucosylation of N-glycans^13^, and core fucosylation disappears after *FUT8* knockout (KO)^27^. We confirmed the absence of core fucose on HepG2-hNTCP-C4 *FUT8* KO cells by confocal microscopy and flow cytometry with PhoSL-FITC (Fig. 3B, C). The level of HBeAg secretion from HepG2-hNTCP-C4 *FUT8* KO cells was lower than that from WT cells after HBV inoculation (Fig. 3D). This difference could be explained by a decrease in HBV particle endocytosis mediated by the heparan sulfate proteoglycan (HSPG) pathway^17^ or the NTCP-EGFR pathway^24^. Core fucosylation increases endocytosis of bio-nano capsules^12^, which can bind to HSPG but not to NTCP, and increases the efficiency of EGFR activation^25^. We inoculated HepG2-hNTCP-C4 WT and *FUT8* KO cells with HBV in the presence or absence of PhoSL for 1 day. After removal of free HBV and PhoSL, the cells were cultured for 9 days. PhoSL decreased the levels of HBeAg secretion from WT and *FUT8* KO cells (Fig. 3D). The level of HBeAg secretion from *FUT8* KO cells treated with PhoSL decreased to approximately 40% of those cells that were not treated with PhoSL. These results indicate that PhoSL can inhibit HBV infection by not only binding to host cells but also by binding to HBV itself, as PhoSL did not bind to HepG2-hNTCP-C4 *FUT8* KO cells.

### HBV bound by PhoSL is internalized into HepG2-hNTCP-C4 WT cells

**Figure 4:**
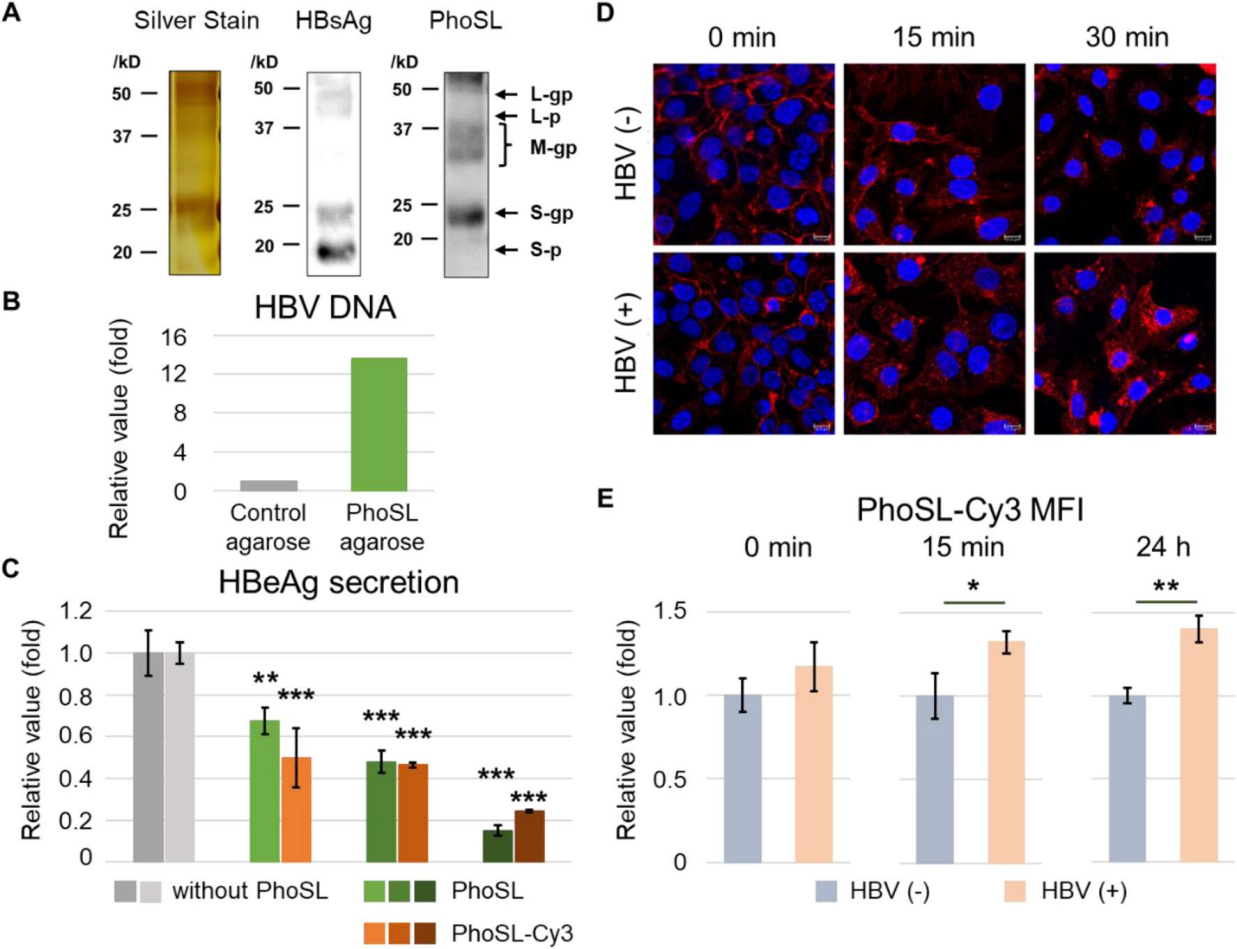
PhoSL bound to HBV particles and HBV-bound PhoSL was incorporated into HepG2-hNTCP-C4 cells. (**A**) Lectin blot of HBsAg purified from the conditioned medium of HepAD38.7 cells. HBsAg was immunoprecipitated with anti-HBsAg polyclonal antibody. Left: silver stain; Center: immunoblot with anti-HBsAg polyclonal antibody; Right: lectin blot with PhoSL. L, M and S indicates Large-HBsAg, Middle-HBsAg and Small-HBsAg, respectively. gp and p indicate glycosylated and no glycosylated form, respectively. The original gel and blot images are available in Supplementary Fig. S6 online. (**B**) qPCR quantification of HBV DNA. PhoSL cross-linked to agarose was mixed at 4 °C for 2 h with conditioned medium from HepAD38.7 cells that contained HBV particles. (**C**) HBeAg secretion from HepG2-hNTCP-C4 cells pretreated with or without PhoSL at 4 °C for 1 h and then inoculated with HBV particles in the absence or presence of PhoSL was measured by ELISA. The increasing gradients of green and orange represent PhoSL and PhoSL-Cy3 concentrations of 2.5, 5, and 10 μg/mL, respectively. Gray indicates 0 μg/mL PhoSL. Dunnett’s test, **P < 0.01, ***P < 0.001 vs. 0 μg/mL PhoSL. Error bars show SD, n = 3. (**D**) Confocal microscopy of PhoSL-Cy3 internalization by HepG2-hNTCP-C4 cells. PhoSL-Cy3 was attached to HepG2-hNTCP-C4 cells with or without HBV particle inoculation at 4 °C for 1 h, and then the cells were incubated at 37 °C for 0, 15, or 30 min to allow incorporation of PhoSL-Cy3. Upper: without HBV; lower: with HBV. Red: PhoSL-Cy3; blue: nucleus (DAPI stain). Scale bars show 10 μm. (**E**) Quantification of flow cytometry mean fluorescence intensities (MFIs) of PhoSL-Cy3 in HepG2-hNTCP-C4 cells with or without HBV inoculation. Student’s t test, *P < 0.05, **P < 0.01 HBV ( −) vs. HBV ( +). Error bars show SD, n = 3.

**Figure 5:**
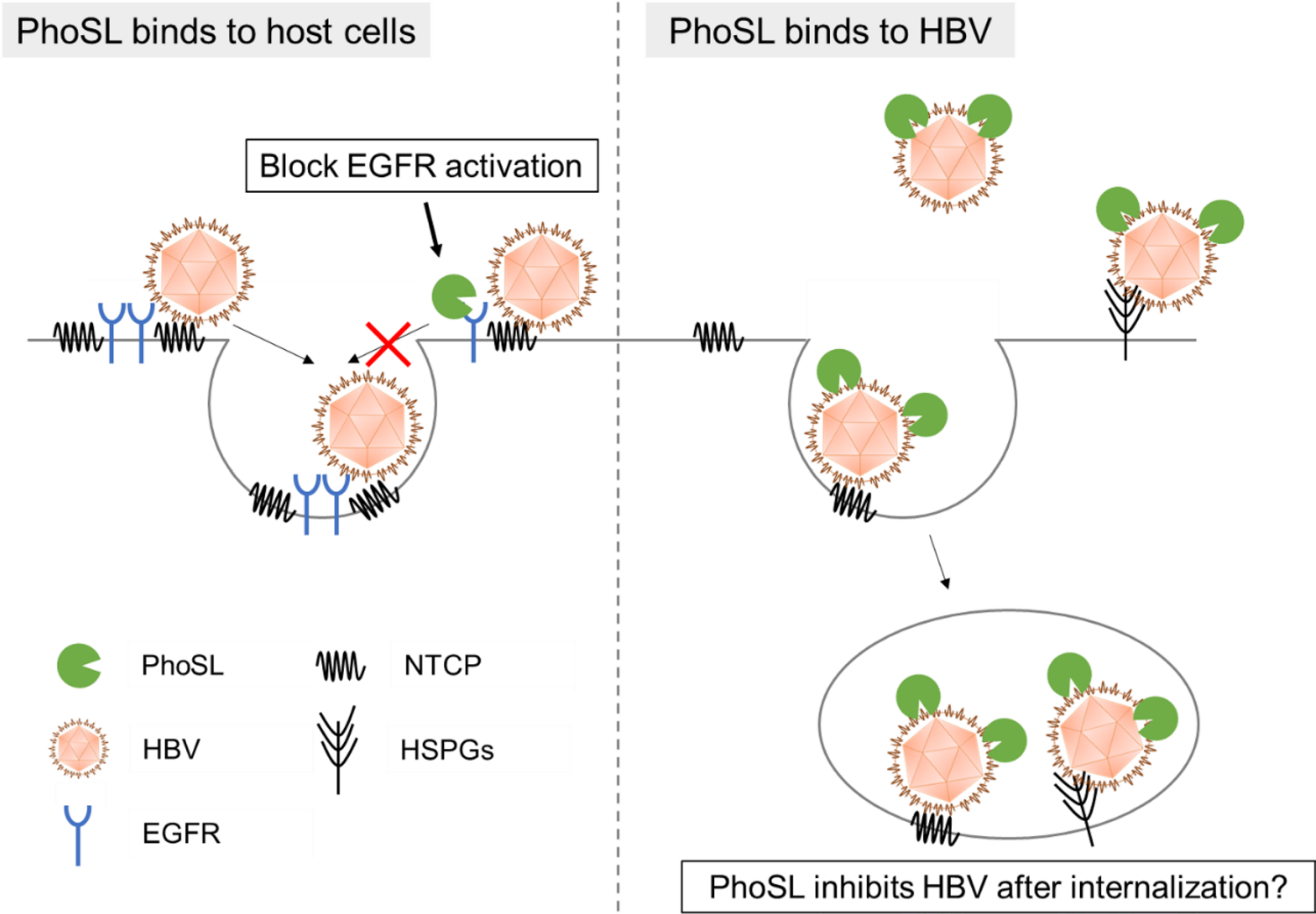
PhoSL inhibits HBV infection by binding to host cells and HBV particles. (Left) PhoSL inhibits EGFR activation by blocking the binding of EGF to EGFR. (Right) PhoSL binds to HBV particles. HBV particles bound by PhoSL are internalized into host cells. PhoSL seems to inhibit HBV infection after internalization.

Finally, we tested the second possibility of how PhoSL inhibits HBV infection: whether PhoSL binds HBV particles. HBV consists of a nucleocapsid, and an envelope that is formed from lipids and three proteins: the large, medium, and small hepatitis B surface antigens (L-HBsAg, M-HBsAg, and S-HBsAg, respectively). These transmembrane proteins have a common carboxy-terminal sequence corresponding to that of the S protein. The S domain is often N-glycosylated^28^. First, to check whether HBsAg in conditioned medium containing HBV particles from HepAD38.7, a cell line that produces HBV particles^29^, was core fucosylated, we performed immunoprecipitation of HBsAg and lectin blot with PhoSL. Lectin blot with PhoSL revealed that HBsAg from HepAD38.7 cells was core fucosylated (Fig. 4A). Next, to examine whether PhoSL binds to HBV particles, we prepared agarose cross-linked to PhoSL (PhoSL-agarose) and mixed it with conditioned medium containing HBV particles from HepAD38.7 cells. PhoSL-agarose captured thirteen times more HBV particles than control agarose, as measured by quantitative polymerase chain reaction (qPCR) (Fig. 4B). Finally, to reveal the significance of PhoSL binding to HBV on HBV infection inhibition, we labeled PhoSL with Cy3 and observed the dynamics of PhoSL-Cy3 on HepG2-hNTCP-C4 cells with or without HBV inoculation. Cy3 labeling did not affect the inhibitory efficiency of HBV infection by PhoSL (Fig. 4C). Because PhoSL inhibited cccDNA formation (Fig. 1C), we investigated whether PhoSL affected HBV dynamics by binding to HBV particles. PhoSL-Cy3 was internalized into HepG2-hNTCP-C4 cells with and without HBV inoculation for up to 30 min at 37 °C (Fig. 4D). Then, HepG2-hNTCP-C4 cells were treated with PhoSL-Cy3 in the presence or absence of HBV particles and were trypsinized, which detaches bound HBV from the cell surface^17^. The mean fluorescence intensities of PhoSL-Cy3 with HBV inoculation were higher than those without HBV inoculation at 37 °C for 15 min and 24 h by flow cytometry (Fig. 4E). These data suggest that HBV particles bound to PhoSL are internalized into HepG2-hNCP-C4 cells, and PhoSL may inhibit HBV infection after the internalization. We used these data to develop a schematic that presents how PhoSL can inhibit HBV infection by binding to host cells and HBV particles (Fig. 5).

## Discussion

In this study, we showed that PhoSL, a lectin that specifically and strongly binds to core fucose, inhibited HBV infection in a dose-dependent manner without cytotoxicity (Fig. 1). Moreover, PhoSL inhibited HBV infection by binding to HepG2-hNTCP-C4 cells and HBV particles (Figs. 2, 3, 4). Glycans on host cells and viruses are important factors for virus infection^9^. Viruses are internalized using the endocytosis machinery of host cells, and changes in glycans can affect that machinery. EGFR, a heavily *N*-glycosylated receptor tyrosine kinase, is a coreceptor for HBV infection, and its activation enhances HBV endocytosis via NTCP^24^, an essential receptor for HBV. EGFR activation by autophosphorylation occurs by the binding of its ligand, EGF, which is followed by receptor dimerization^30^. EGFR is *N*-glycosylated around the EGF binding site^31^. EGFR binds EGF with two distinct affinities: high affinity (*K*d $\approx$ 300 pM) and low affinity (*K*d $\approx$ 2 nM)^32^. Core fucosylation affects high-affinity binding of EGF to EGFR^25^, indicating that *N*-glycans with core fucose are important regions of EGFR that affect EGF binding. In Supplementary Fig. S2 online, PhoSL (10 μg/mL) blocked the binding of EGF-TAMRA (100 ng/mL or ≈17 nM) to EGFR. Therefore, PhoSL seems to prevent EGF binding to EGFR by steric hindrance.

We also found that PhoSL binds to HBV particles (Fig. 4B). HBV is an enveloped virus, and the envelope consists of lipids and proteins, including HBsAg. HBsAg in conditioned medium containing HBV particles from HepAD38.7 cells was core fucosylated (Fig. 4A). Therefore, PhoSL seems to bind to HBV by recognizing core fucose of HBsAg. Cyanovirin-N, a lectin produced by the cyanobacterium *Nostoc ellipsosporum*, binds to gp120, a protein on the HIV envelope, and inhibits HIV infection by HIV membrane fusion^33,34^. Recently, it was reported that PhoSL inhibits SARS-CoV-2 infection by binding to S protein, a protein on the SARS-CoV-2 envelope^35^. We found that PhoSL inhibited HBV infection of HepG2-hNTCP-C4 *FUT8* KO cells, which cannot bind PhoSL (Fig. 3D). Thus, PhoSL may inhibit HBV infection by binding to HBV particles. The early phase of HBV infection consists of HBV attachment, endocytosis, membrane fusion, and cccDNA formation^36^. To reveal the significance of PhoSL binding to HBV particles on the inhibition of HBV infection, we investigated PhoSL-Cy3 dynamics with HBV inoculation. Flow cytometry analysis showed that the signals from PhoSL-Cy3 increased in the presence of HBV particles (Fig. 4E). Because trypsinization detaches HBV particles from cell surfaces^17^, the increase in PhoSL-Cy3 seems to indicate that PhoSL-bound HBV particles can be internalized . These data suggest that PhoSL-bound HBV particles might inhibit processes after the endocytosis. Possible mechanisms responsible for PhoSL-mediated inhibition of HBV infection were described in Fig. 5. PhoSL treatment could contribute to the development of novel anti-HBV therapies.

## Materials and methods

### Reagents

The reagents used are as follows: Williams E medium (Gibco or Sigma), fetal bovine serum (Sigma or Nichirei Biosciences), penicillin–streptomycin mixed solution (stabilized; Nacalai Tesque), GlutaMAX-I (Gibco), L-glutamine (Nacalai Tesque), insulin-transferrin-selenium (Gibco), holo-transferrin (FUJIFILM Wako), insulin (Sigma), hydrocortisone (Sigma or Nacalai Tesque), dexamethasone (Sigma), EGF (Thermo Fisher Scientific or PeproTech), G418 (FUJIFILM Wako), EGF-TAMRA (Thermo Fisher Scientific), Hoechst 33,342 (Nacalai Tesque), dimethyl sulfoxide (DMSO; FUJIFILM Wako), polyethylene glycol 8000 (PEG 8000; MP Biomedicals), and the HBeAg enzyme-linked immunosorbent assay (ELISA) kit (Bioneovan or Shanghai Rongsheng Biotech).

### Antibodies and lectins

The antibodies used are as follows: anti-EGFR monoclonal antibody (#ab52894, Abcam), anti-phospho-EGFR (Tyr1068) monoclonal antibody (#2234, Cell Signaling Technology), and anti-β-actin-horseradish peroxidase (HRP) monoclonal antibody (#5125, Cell Signaling Technology) and anti-HBsAg polyclonal antibody (for immunoprecipitation; #bs-1557G, Bioss, for immunoblot; #BCL-ABPS-01, Beacle).

PhoSL, PhoSL-fluorescein (FITC), and PhoSL-biotin were provided by Dr. Kobayashi of J-Chemical. Streptavidin-FITC (#SA-5001) was purchased from Vector Laboratories.

### Cells

HepG2-hNTCP-C4 WT cells were obtained from the National Institute of Infectious Diseases (Tokyo, Japan)^37^ and maintained in primary hepatocyte maintenance medium (PMM), which consisted of Williams E medium supplemented with 10% heat-inactivated fetal bovine serum; 1% penicillin–streptomycin mixed solution (stabilized) or a mixture of penicillin (100 units/mL), streptomycin (100 µg/mL), and amphotericin B (0.025 µg/mL); GlutaMAX-I (2 mM) or L-glutamine (2 mM); 1 × insulin-transferrin-selenium or a mixture of transferrin (5 µg/mL), human insulin (3 µg/mL), and sodium selenite (5 ng/mL); hydrocortisone (50 µM); dexamethasone (5 µM); EGF (10 ng/mL); and G418 (0.5 mg/mL). For the PhoSL inhibition assay to measure EGFR activation, HepG2-hNTCP-C4 WT cells were maintained in Dulbecco’s modified Eagle medium/Nutrient Mixture F-12 medium (DMEM/F-12) supplemented with 10% heat-inactivated fetal bovine serum, 1% penicillin–streptomycin mixed solution (stabilized), GlutaMAX-I (2 mM), and HEPES (10 mM).

The HepG2-hNTCP-C4 *FUT8* KO cell line was established by a CRISPR-Cas9 system. HepG2-hNTCP-C4 WT cells were transfected with the PX462 plasmid (Addgene) containing h*FUT8* single guide RNA (sgRNA) using Lipofectamine 2000 (Invitrogen) and selected with puromycin (2 μg/mL) for 24 h. After medium replacement, live cells were transferred from six-well plates to 150-mm dishes and cultured for a further two weeks. KO clones were established by isolating colonies with a cloning cylinder. KO was confirmed by genome sequencing. The sgRNAs used are listed in Table 1.

**Table 1:** sgRNAs for human *FUT8* KO in HepG2-NTCP-C4 cells.

h*FUT8* sgRNA sense 1	5′-caccGAATGAGCATAATCCAACGCC-3′
h*FUT8* sgRNA antisense 1	5′-aaacGGCGTTGGATTATGCTCATTC-3′
h*FUT8* sgRNA sense 2	5′-caccGACCTTGCTGTTTTATATAGG-3′
h*FUT8* sgRNA antisense 2	5′-aaacCCTATATAAAACAGCAAGGTC-3′

HepAD38.7 cells, a tetracycline-regulated HBV-producing cell line that was established using HepG2 cells^29^, were kindly provided by Dr. Christoph Seeger at Fox Chase Cancer Center (Philadelphia, PA, USA) and were maintained in DMEM/F12 medium supplemented with 10% heat-inactivated fetal bovine serum, 1% antibiotic–antimycotic solution, insulin (5 µg/mL), G418 (0.5 mg/mL), and tetracycline (400 ng/mL).

PANC-1 cells were obtained from RIKEN BioResource Research Center and were maintained in RPMI 1640 medium supplemented with 10% heat-inactivated fetal bovine serum, 1% penicillin–streptomycin mixed solution (stabilized), and GlutaMAX-I (2 mM).

### Preparation of HBV particles

To obtain HBV particles for HBV infection experiments, HepAD38.7 cells were induced for HBV replication by omitting tetracycline from the culture medium. The culture medium of confluent HepAD38.7 cells grown without tetracycline was collected each week for 2 weeks, and HBV particles were precipitated from the medium with PEG 8000 (final concentration 6%) at 4 °C overnight. The precipitate was pelleted by centrifugation, and the precipitated HBV particles were resuspended in phosphate-buffered saline (PBS), concentrated, and filtered through a 0.45-µm filter (Millipore). The HBV DNA was quantified by qPCR.

### HBV infection of HepG2-hNTCP-C4 cells

HepG2-hNTCP-C4 cells were seeded on type I collagen-coated 24-well plates (Iwaki) at 0.5 or 1 × 10^5^ cells/well and cultured for 1 day at 37 °C under humidified conditions and 5% CO_2_. The cells were then infected with HBV particles at 1,000 genome equivalents of infection (GEI) together with PhoSL (0, 0.5, 1, 2.5, 5, or 10 μg/mL) with or without gefitinib (10 μM) in PMM (0.5 mL) containing 2% DMSO and 4% PEG 8000. After incubation for 24 h, the cells were washed twice with PMM containing 2% DMSO and were cultured in PMM (0.5 mL) containing 2% DMSO for 9 days. Finally, the supernatant and cells were harvested for analysis.

### HBeAg ELISA

HBeAg in supernatants from HepG2-hNTCP-C4 cells with or without HBV infection was measured using a commercially available HBeAg ELISA kit. The absorbances were measured at 450 nm and 630 nm on a SpectraMax 190 microplate reader (Molecular Devices) or an SH-1200Lab microplate reader (Corona Electric).

### qPCR

HBV DNA was extracted as follows. Cells were lysed with hypotonic buffer (20 mM Tris–HCl pH 7.5–7.8, 50 mM NaCl, 5 mM MgCl_2_, and 0.1% 2-mercaptoethanol), and the supernatant was treated with 1% sodium dodecyl sulfate and proteinase K (0.2 mg/mL) overnight at 56 °C. After incubation, the DNA was extracted with phenol–chloroform-isoamyl alcohol and purified by ethanol precipitation.

For cccDNA preparation, cell pellets were lysed by resuspension in a hypotonic buffer of 1 × TE buffer containing 1% sodium dodecyl sulfate. Then, NaCl (0.5 M) was added, and the lysate was incubated overnight at 4 °C. After centrifugation, the supernatant was treated with proteinase K (0.2 mg/mL). DNA was extracted from the supernatant with phenol–chloroform-isoamyl alcohol and purified by ethanol precipitation. To isolate the cccDNA, purified DNA was digested with Plasmid-Safe ATP-Dependent DNase (Lucigen) and purified by phenol–chloroform-isoamyl alcohol extraction and ethanol precipitation.

HBV RNA was isolated with TRIzol (Invitrogen), and cDNA was synthesized by reverse transcription. qPCR was performed using a QuantStudio 6 Flex Real-Time PCR System (Applied Biosystems). The primers used are listed in Table 2.

**Table 2:** Primers for qPCR of HBV DNA, cccDNA, and RNA.

HBV DNA forward primer	5′-CTTCATCCTGCTGCTATGCCT-3′
HBV DNA reverse primer	5′-AAAGCCCAGGATGATGGGAT-3′
cccDNA forward primer	5′-GTCTGTGCCTTCTCATCTGC-3′
cccDNA reverse primer	5′-GCACAGCTTGGAGGCTTGAA-3′
HBV RNA forward primer	5′-ATCATCTCATGTTCATGTCCTAC-3′
HBV RNA reverse primer	5′-GGAGATCTCGAATAGAAGGAAAG-3′

Ref. ^38,40^.

### Cytotoxicity assay

HepG2-hNTCP-C4 WT cells were seeded on a type I collagen-coated 96-well plate (Iwaki) at 3,000 cells/well and cultured for 1 day at 37 °C under humidified conditions and 5% CO_2_. After changing to fresh medium, cells were cultured with PhoSL (0, 0.25, 0.5, 1, 2.5, 5, or 10 μg/mL) for 24 h. Viability was measured using a CellTiter-Glo Luminescent Cell Viability Assay kit (Promega), and chemiluminescence intensity was measured with an SH-9000 microplate reader (Corona Electric).

### PhoSL inhibition assay to measure EGFR activation

HepG2-hNTCP-C4 WT and PANC-1 cells were seeded on type I collagen-coated 60-mm dishes or type I collagen-coated 6-well plates (Iwaki) and cultured at 37 °C under humidified conditions and 5% CO_2_. The cells were starved for 1 day by changing to a serum-free medium. Then, gefitinib (10 μM) as a positive control or PhoSL (10 μg/mL) was added to the medium 3 h or 10 min before stimulation with EGF (100 ng/mL) or EGF-TAMRA (100 ng/ml). The cells were incubated for 10 min at 37 °C under humidified conditions and 5% CO_2_, washed with PBS, and harvested with cell scrapers in ice-cold TNE buffer (10 mM Tris–HCl pH 7.8, 1% NP-40, 1 mM EDTA, and 0.5 M NaCl) containing 1 × protease inhibitor cocktail and 1 × phosphatase inhibitor cocktail 2. After 15 min incubation on ice, the lysates were sonicated with a Bioruptor UCD-200 T (Cosmo Bio) and centrifugated at 20,000 × *g* for 20 min at 4 °C. The supernatants were collected as lysate samples for immunoblot.

### Immunoblot

Cell lysates were subjected to 10% sodium dodecyl sulfate–polyacrylamide gel electrophoresis under reducing conditions and subsequently transferred to polyvinylidene fluoride membranes (Millipore). After blocking with Tris-buffered saline with 0.05% Tween 20 (TBST) containing 5% skim milk powder for 1 h at room temperature, the membranes were incubated with a primary antibody in TBST containing 5% skim milk powder overnight at 4 °C. The incubations for the anti-phospho-EGFR primary antibody were performed in TBST containing 5% bovine serum albumin. After three washes with TBST (each for 10 min at room temperature), the membranes were incubated with anti-rabbit IgG-HRP polyclonal secondary antibody (1:10,000 dilution) in TBST containing 5% skim milk powder for 1 h at room temperature. After washing with TBST three times (each for 10 min at room temperature), the HRP signal was detected with Chemi-Lumi One Super (Nacalai Tesque) and imaged with a Fusion Solo S system (Vilber). Band intensities were quantified with Fiji software (https://fiji.sc/). Primary antibody dilution ratios are listed in Table 3.

**Table 3:** Primary antibodies and dilution ratios for immunoblots.

Anti-EGFR monoclonal antibody	1:3000
Anti-phospho-EGFR (Tyr1068) monoclonal antibody	1:1000
Anti-β-actin-HRP monoclonal antibody	1:10,000
Anti-HBsAg polyclonal antibody	1:1000

### Silver stain and Lectin blot with PhoSL

We immunoprecipitated HBsAg with anti-HBsAg polyclonal antibody cross-linked Protein G Sepharose. We cross-linked anti-HBsAg polyclonal antibody with Protein G Sepharose 4 Fast Flow (GE Healthcore) as described below. Anti-HBsAg polyclonal antibody (10 μg; Bioss) was mixed with Protein G Sepharose 4 Fast Flow (40 μl) in TBST for 2 h at 4 °C. After washing with TBST and 200 mM triethanolamine (pH 8.9), cross-linked Sepharose was mixed with 50 mM dimethyl pimelimidate dihydrochloride in 200 mM triethanolamine (pH 8.9) for 1 h at room temperature. To quench unreacted DMP, after washing 200 mM triethanolamine (pH 8.9), cross-linked Sepharose was rotated with 100 mM ethanolamine (pH 8.9) for 15 min at room temperature. Antibodies not cross-linked was removed with 0.1 M glycine (pH 2.9) and 2 M urea. Finally, cross-linked Sepharose was washed with solubilization buffer (10 mM Tris–HCl pH 7.3, 0.5 M NaCl and 2% Triton X-100) three times.

We immunoprecipitated HBsAg as described below. To solubilize HBV particles, HBV particles prepared from the conditioned medium of HepAD38.7 cells were mixed with solubilization buffer and incubated at 37 °C overnight^39^. After preclearing with Protein G Sepharose, cross-linked Sepharose was rotated with solubilized HBV particles solution at 4 °C overnight. The Sepharose beads were washed with TBST at three times and then boiled with sample buffer (50 mM Tris–HCl (pH 6.8), 2% sodium dodecyl sulfate, 10% glycerol, 0.025% bromophenol blue and 5% 2-mercaptoethanol) for 5 min at 95 °C.

Immunoprecipitated HBsAg subjected to 15% sodium dodecyl sulfate–polyacrylamide gel electrophoresis under reducing conditions. Silver stain was performed with Silver Stain MS Kit (FUJIFILM Wako) according to the manufacturer’s protocol. We performed lectin blot analysis with PhoSL as described below. After transferred to polyvinylidene fluoride membranes (Millipore), the membranes was blocked with TBST containing 3% bovine serum albumin at 4 °C overnight. The membranes were incubated with PhoSL-biotin (1:2,000 dilution) in TBST containing 3% bovine serum albumin for 1 h at room temperature. After three washes with TBST (each for 5 min at room temperature), the membranes were incubated with Streptavidin-HRP (1:10,000 dilution) in TBST containing 3% bovine serum albumin for 1 h at room temperature. After washing with TBST three times (each for 5 min at room temperature), the HRP signal was detected with Chemi-Lumi One Super (Nacalai Tesque) and imaged with a Fusion Solo S system (Vilber).

### Preparation of PhoSL-Cy3

We labeled PhoSL (100 μg at 1 mg/mL) with Cy3 Mono-Reactive Dye (100 μg; Cytiva) in PBS. After 1 h incubation at room temperature, free Cy3 was removed using Zeba Spin Desalting Columns (Thermo Fisher Scientific).

### Fluorescence staining

HepG2-hNTCP-C4 WT and *FUT8* KO cells were seeded at 6 × 10^5^ cells on type I collagen-coated glass-based dishes (Iwaki) and cultured for 1 day at 37 °C under humidified conditions and 5% CO_2_. After washing with PBS, the cells were fixed with 4% paraformaldehyde in PBS for 15 min at room temperature. After three washes with PBS (each for 5 min at room temperature), the cells were stained with PhoSL-FITC (1:10,000 dilution) and Hoechst 33,342 (1:1,000 dilution) in PBS containing 2% bovine serum albumin for 1 h at 4 °C. After washing with PBS three times (each for 5 min at room temperature), the cells were observed and imaged with a FLUOVIEW FV10i confocal laser-scanning microscope (Olympus).

For the EGF-TAMRA binding assay, the cells were starved for 1 day by changing to a serum-free medium. PhoSL (10 μg/mL) was added 10 min before stimulation with EGF-TAMRA (100 ng/mL). After 10 min incubation at 37 °C under humidified conditions and 5% CO_2_, the cells were washed with PBS and fixed with 4% paraformaldehyde.

For PhoSL-Cy3 observation, HepG2-hNTCP-C4 WT cells were seeded at 1 × 10^4^ cells/well on collagen-coated eight-well chamber slides (Matsunami Glass) and incubated at 37 °C under humidified conditions and 5% CO_2_ for 3 days. The cells were then infected with HBV at 2,000 GEI in PMM (0.5 mL) containing 2% DMSO, 4% PEG 8000, and PhoSL-Cy3 (10 μg/mL). After washing with PBS, the cells were fixed with 4% paraformaldehyde, and the slides were coverslipped with Fluoro-KEEPER Antifade Reagent, Non-Hardening Type with 4′,6-diamidino-2-phenylindole (DAPI) (Nacalai Tesque). The cells were observed and imaged with a TCS SP8 confocal microscope (Leica).

### Flow cytometry

To confirm core fucose depletion, HepG2-hNTCP-C4 WT and *FUT8* KO cells were detached from cell culture dishes with accutase (Nacalai Tesque) and centrifuged at 200 × *g* for 5 min at 4 °C. After washing with PBS, the cells were stained with PhoSL-FITC (1:10,000 dilution) in PBS containing 1% bovine serum albumin for 1 h at 4 °C. After washing twice with PBS, flow cytometry was performed with an Attune NxT Flow Cytometer (Thermo Fisher Scientific). Supplementary Fig. S2 online shows the results of staining of HepG2-hNTCP-C4 WT and PANC-1 cells with PhoSL-biotin (1:100 dilution) and streptavidin-FITC (1:100 dilution).

For quantification of PhoSL-Cy3 staining, HepG2-hNTCP-C4 WT cells were seeded at 6 × 10^5^ cells/well on type I collagen-coated six-well plates (Iwaki) and cultured for 1 day at 37 °C under humidified conditions and 5% CO_2_. Then, cells were infected with HBV at 2,000 GEI in PMM containing 2% DMSO and 4% PEG 8000 in the presence of PhoSL-Cy3 (5 μg/mL). After 1 h incubation at 4 °C, cells were placed at 37 °C and trypsinized after 0 min, 15 min, or 24 h. Flow cytometry was performed using a BD FACSCanto II Flow Cytometer (BD Biosciences). Data were analyzed with FlowJo (https://www.flowjo.com/).

### PhoSL-mediated precipitation of HBV particles

PhoSL was cross-linked to Pierce NHS-Activated Agarose Dry Resin (Thermo Fisher Scientific) in accordance with the manufacturer’s protocol. Next, the PhoSL-agarose was rotated with the conditioned medium containing HBV particles isolated from HepAD38.7 cells for 2 h at 4 °C. After washing with Tris-buffered saline, DNA was extracted from the PhoSL-agarose, and qPCR was performed using a QuantStudio 6 Flex Real-Time PCR System (Applied Biosystems).

### Statistical analysis

All statistical analyses were performed with EZR software^40^ (https://www.jichi.ac.jp/saitama-sct/SaitamaHP.files/statmedEN.html), and data were presented as mean ± standard deviation (SD) where applicable. Dunnett’s test and Student’s t test with Bonferroni correction were used.

## Supplementary Information

Supplementary Information.

## Data Availability

The data presented in this study are available from the corresponding author upon reasonable request. The original blot images are available in the Supplementary Information figures.
