# Supplementary material for: The specific core fucose-binding lectin Pholiota squarrosa lectin (PhoSL) inhibits hepatitis B virus infection in vitro
Source: Sci Rep. 2023 Apr 15;13:6175. doi: 10.1038/s41598-023-28572-6 (PMC10105536; doi:10.1038/s41598-023-28572-6)
Supplement: Supplementary file 1 — Supplementary Information. [file 41598_2023_28572_MOESM1_ESM.pdf]

# ***The specific core fucose-binding lectin Pholiota squarrosa lectin (PhoSL) inhibits hepatitis B virus infection in vitro***

Tsunenori Ouchida[1, 2, #], Haruka Maeda[1, 3, #], Yuka Akamatsu[1, 3], Megumi Maeda[1, 3], Shinji Takamatsu[1], Jumpei Kondo[1], Ryo Misaki[4], Yoshihiro Kamada[5], Masahiro Ueda[2], Keiji Ueda[3], and Eiji Miyoshi[1, *]

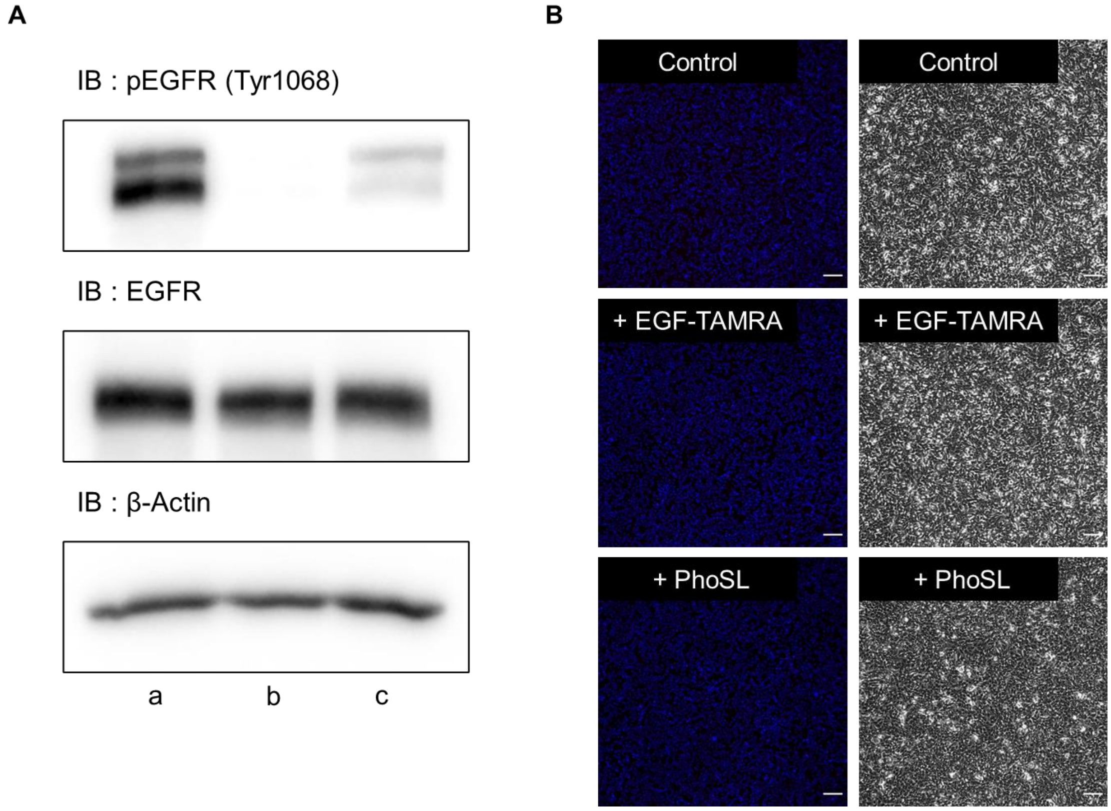


## Supplementary Fig. S1. PhoSL blocked EGFR activation on HepG2-hNTCP-C4 cells as measured by EGF-TAMRA

(A) After starvation in serum-free medium, HepG2-hNTCP-C4 cells were stimulated with EGF-TAMRA (100 ng/mL) with gefitinib (10 μM) or PhoSL (10 μg/mL) at 37°C for 10 min. Phosphorylated EGFR in cell lysates was detected by immunoblot. a: no inhibitor; b: gefitinib (10 μM); c: PhoSL (10 μg/mL). The upper band in IB: anti-phospho-EGFR (Tyr1068) monoclonal antibody did not appear to be EGFR. This anti-phospho-EGFR (Tyr1068) monoclonal antibody (#2234, Cell Signaling Technology) may cross-react with other activated EGFR family members (e.g., Erb-B2 receptor tyrosine kinase 2) and cross-reacts slightly with activated platelet-derived growth factor receptor. The original blot images are available in Supplementary Fig. S4.

(B) After starvation in serum-free medium, HepG2-hNTCP-C4 cells were stimulated with EGF-TAMRA (100 ng/mL) with PhoSL (10 μg/mL) at 37°C for 10 min. Then the cells were fixed and observed using confocal microscopy. Red: EGF-TAMRA; blue: nucleus (Hoechst 33342 stain). Bright-field images are shown on the right. Scale bars indicate 100 μm.

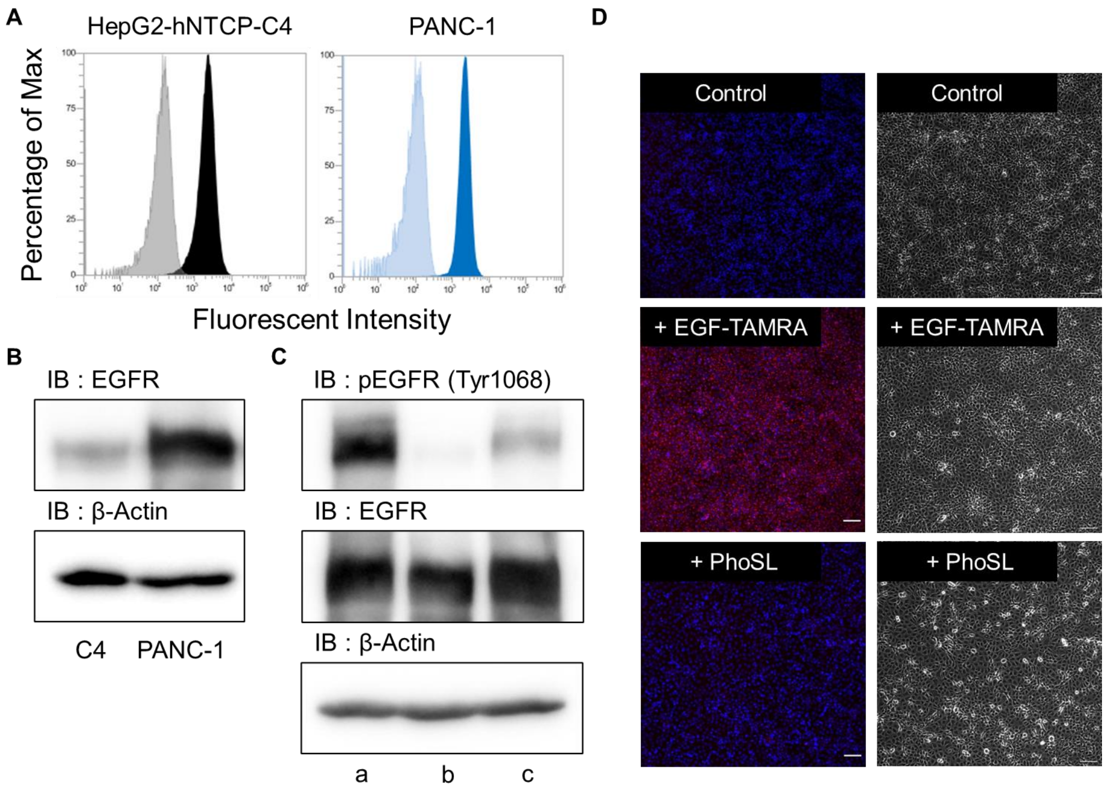


## Supplementary Fig. S2. PhoSL blocked EGF-TAMRA binding to EGFR.

(A) Core fucosylation of PANC-1 cells. Black: HepG2-hNTCP-C4 WT cells; blue: PANC-1 cells. The lighter colors (left peaks) show the cells stained with streptavidin-FITC (control).

(B) EGFR expression levels of HepG2-hNTCP-C4 and PANC-1 cells. The original blot images are available in Supplementary Fig. S5A, D.

(C) After starvation in serum-free medium, PANC-1 cells were stimulated with EGF-TAMRA (100 ng/mL) with gefitinib (10 μM) or PhoSL (10 μg/mL) at 37°C for 10 min. Phosphorylated EGFR in cell lysates was detected by immunoblot. a: no inhibitor; b: gefitinib (10 μM); c: PhoSL (10 μg/mL). The original blot images are available in Supplementary Fig. S5B, C, and D.

(D) After starvation in serum-free medium, PANC-1 cells were stimulated with EGF-TAMRA (100 ng/mL) with PhoSL (10 μg/mL) at 37°C for 10 min. Then the cells were fixed and observed using confocal microscopy. Red: EGF-TAMRA; blue: nucleus (Hoechst 33342 stain). Bright-field images are shown on the right. Scale bars show 100 μm.

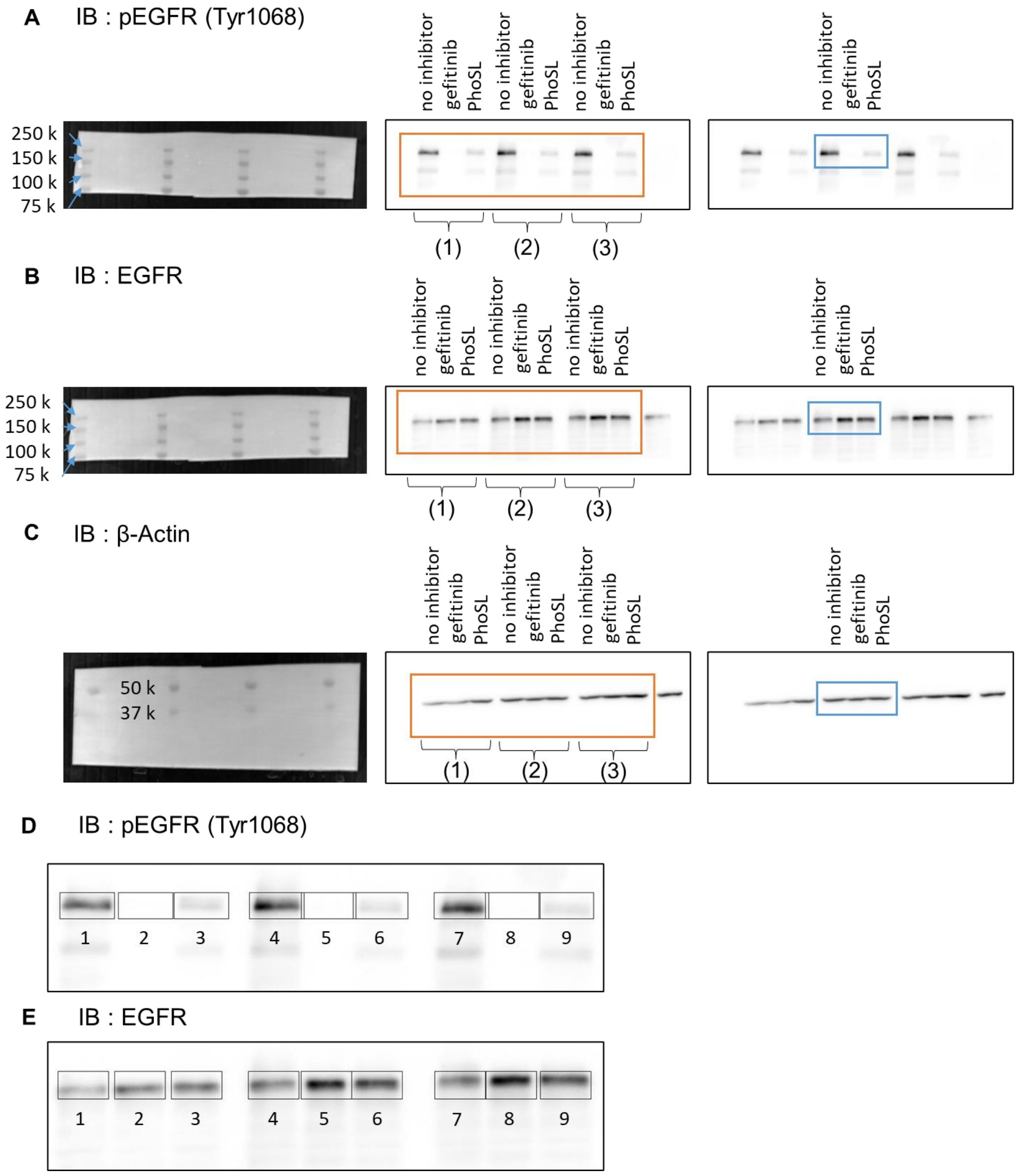


# Supplementary Fig. S3. Uncropped blot images related to Fig. 2B, C.

(A, B, C) Blot images of HepG2-hNTCP-C4 WT cells stimulated with EGF and detected with anti-phospho-EGFR (Tyr1068) monoclonal antibody, anti-EGFR monoclonal antibody, and anti-β-actin monoclonal antibody, respectively. Left: size markers; center and right: blot images. Orange areas show signals related to our data analysis. Blue areas were cropped for Fig. 2B. (1), (2), and (3) were independent experiments from cell seeding to lysate collection. (B) was blotted after stripping (A). These membranes were cut from full-length membranes.

(D, E) The areas marked in boxes were quantified using Fiji software, and the data are shown in Fig. 2C. The signals of phosphorylated EGFR bands were divided by those of corresponding EGFR bands to obtain relative values.

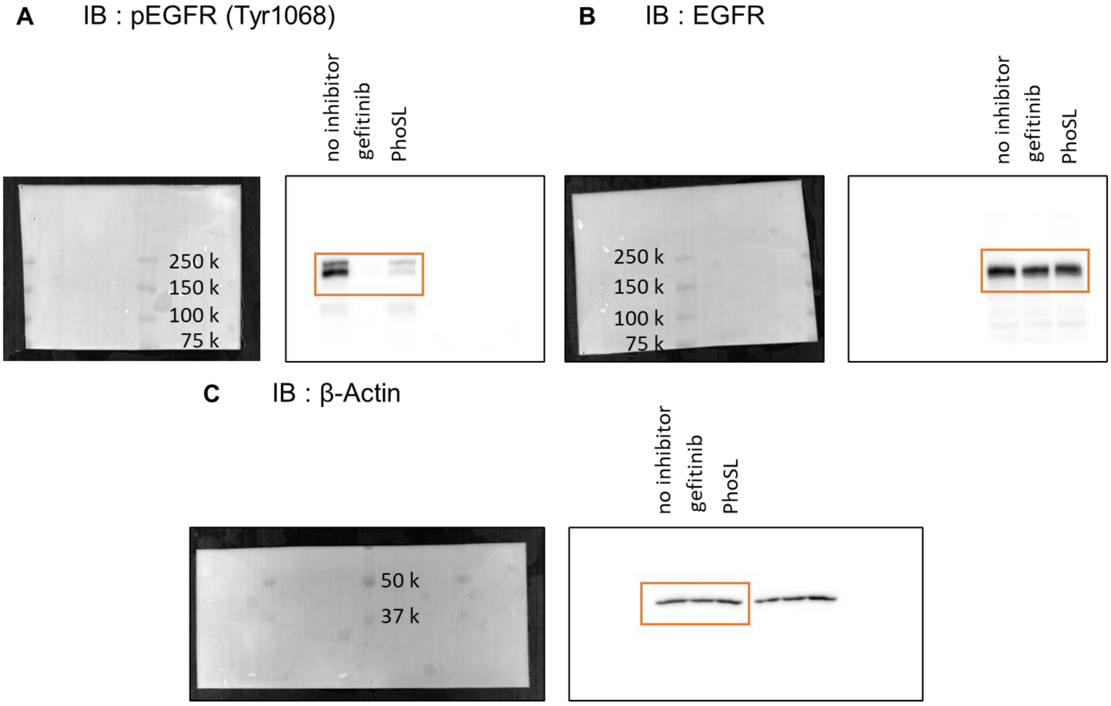


Supplementary Fig. S4. Uncropped blot images related to Supplementary Fig. 1A.

(A, B, C) Blot images of HepG2-hNTCP-C4 WT cells stimulated with EGF-TAMRA and detected with anti-phospho-EGFR (Tyr1068) monoclonal antibody, anti-EGFR monoclonal antibody, and anti-β-actin monoclonal antibody, respectively. Left: size markers; right: blot images. Orange areas were cropped for Supplementary Fig. 1A. These membranes were cut from full-length membranes.

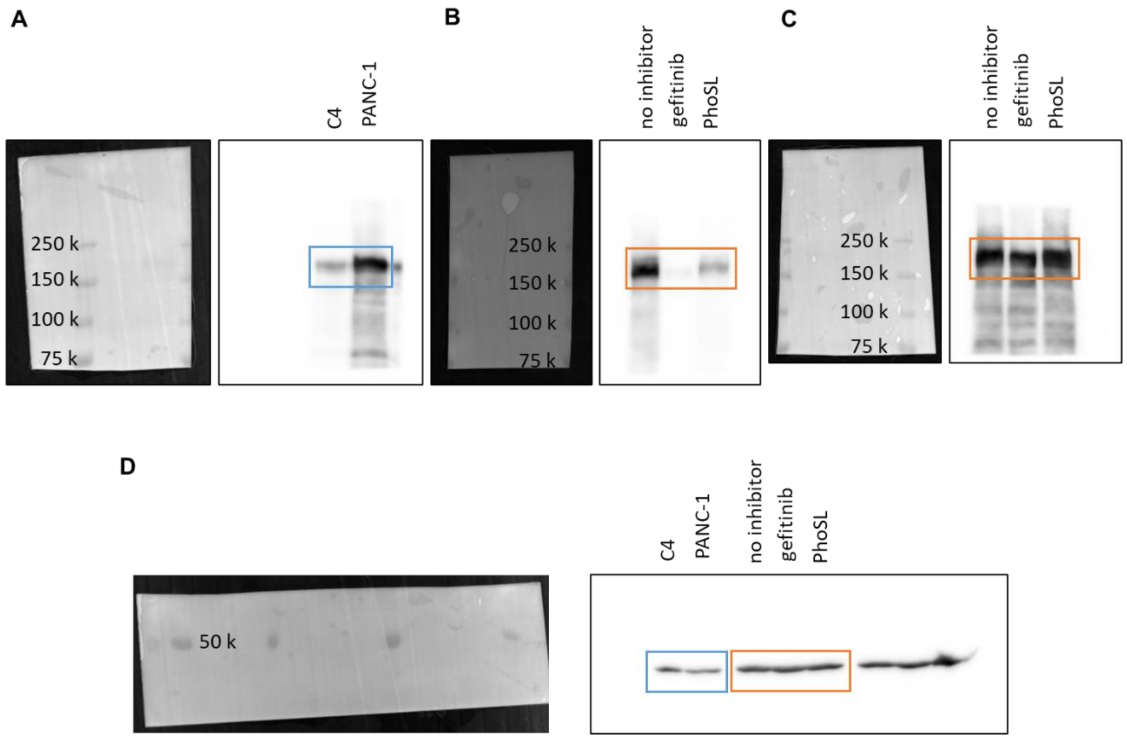


Supplementary Fig. S5. Uncropped blot images related to Supplementary Fig. 2B, C.

(A) Blot images of HepG2-hNTCP-C4 WT and PANC-1 cells detected with anti-EGFR monoclonal antibody. Left: size markers; right: blot images. The blue area was cropped for Supplementary Fig. 2B.

(B, C, D) Blot images of PANC-1 cells detected with anti-phospho-EGFR monoclonal antibody, anti-EGFR monoclonal antibody, and anti-β-actin monoclonal antibody, respectively. Left: size markers; right: blot images. Orange areas were cropped for Supplementary Fig. 2C. In (D), the blue area was cropped for Supplementary Fig. 2B. These membranes were cut from full-length membranes.

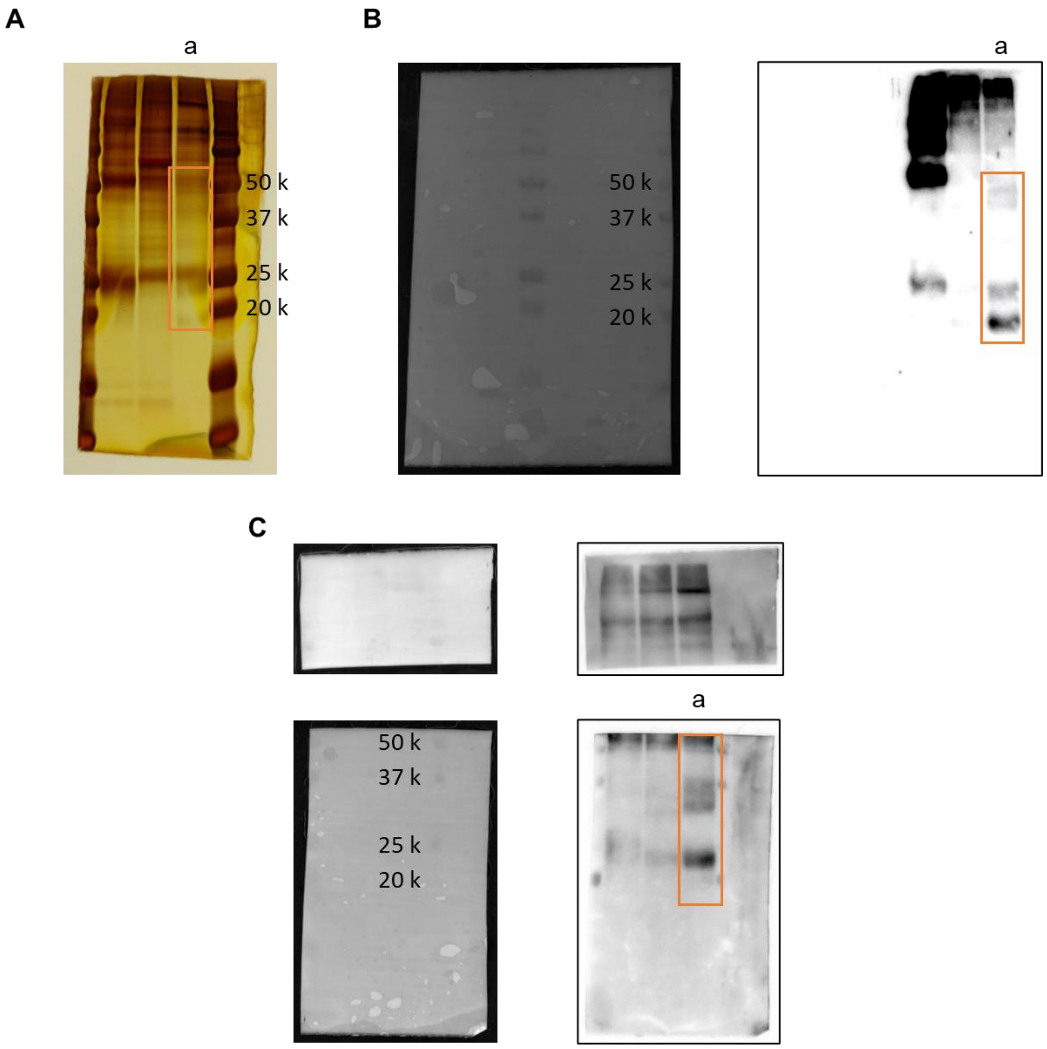


Supplementary Fig. S6. Uncropped gel and blot images related to Fig. 4A.

(A) Gel images of silver stain of immunoprecipitation product with anti-HBsAg polyclonal antibody. Lane a was this experimental product. Left: size markers; right: blot images. The orange area was cropped for Fig. 4A.

(B, C) Blot images of immunoprecipitation product detected with anti-HBsAg polyclonal antibody, and PhoSL, respectively. Left: size markers; right: blot images. Orange areas were cropped for Fig. 4A. Lane a was this experimental product. These membranes were cut from full-length membranes. In lectin blot with PhoSL, because strong signals from molecules >50 kDa interfered, we cut the membrane.
